# Expression of Xenobiotic Metabolizing Cytochrome P450 Genes in a Spinosad-Resistant *Musca domestica* L. Strain

**DOI:** 10.1371/journal.pone.0103689

**Published:** 2014-08-28

**Authors:** Dorte H. Højland, Karl-Martin Vagn Jensen, Michael Kristensen

**Affiliations:** Department of Agroecology, Aarhus University, Slagelse, Denmark; University of Crete, Greece

## Abstract

**Background:**

Spinosad is important in pest management strategies of multiple insect pests. However, spinosad resistance is emerging in various pest species. Resistance has in some species been associated with alterations of the target-site receptor, but in others P450s seems to be involved. We test the possible importance of nine cytochrome P450 genes in the spinosad-resistant housefly strain 791spin and investigate the influence of spinosad on P450 expression in four other housefly strains.

**Results:**

Significant differences in P450 expression of the nine P450 genes in the four strains after spinosad treatment were identified in 40% of cases, most of these as induction. The highly expressed *CYP4G2* was induced 6.6-fold in the insecticide susceptible WHO-SRS females, but decreased 2-fold in resistant 791spin males. *CYP6G4* was constitutively higher expressed in the resistant strain compared to the susceptible strain. Furthermore, *CYP6G4* gene expression was increased in susceptible WHO-SRS flies by spinosad while the expression level did not alter significantly in resistant fly strains. Expression of *CYP6A1* and male *CYP6D3* was constitutively higher in the resistant strain compared to the susceptible. However, in both cases male expression was higher than female expression.

**Conclusion:**

*CYP4G2*, *CYP6A1*, *CYP6D3* and *CYP6G4* have expressions patterns approaching the expectations of a hypothesized sex specific spinosad resistance gene. *CYP4G2* fit requirements of a spinosad resistance gene best, making it the most likely candidate. The overall high expression level of *CYP4G2* throughout the strains also indicates importance of this gene. However, the data on 791spin are not conclusive concerning spinosad resistance and small contributions from multiple P450s with different enzymatic capabilities could be speculated to do the job in 791spin. Differential expression of P450s between sexes is more a rule than an exception. Noteworthy differences between spinosad influenced expression of P450 genes between a field population and established laboratory strains were shown.

## Introduction

Spinosad is a mixture of two macrocyclic lactones; spinosyn A and spinosyn D isolated from the actinomycete bacteria, *Saccharopolyspora spinosa* and has been developed as a commercial insecticide [1]. Its mode of action is unique as its primary target site appears to be a subtype of the nicotinic acetylcholine receptors (nAChRs) with also a secondary target site suggested to be the γ-aminobutyric acid (GABA)-gated chloride channel [2]. Spinosad has been introduced for insect control initially in 1997 and plays an essential role for the control of Lepidoptera and Diptera pests as well as planthoppers, spider mites, ticks, fleas and head lice. There are more than 30 examples of resistance to spinosad and half of these were selected in the field [3]. The mechanism of spinosad resistance has been implied to involve changes of target site and metabolism [4].

A key element in preventing development of resistance as well as resistance management is the understanding of the molecular mechanisms potentially responsible for resistance. Prior to the introduction a decade ago of bait-formulated spinosad for housefly control in Denmark we established a baseline for spinosad toxicity of Danish houseflies. Field populations of houseflies collected from livestock farms throughout Denmark showed up to 7.5-fold resistances (compared to the susceptible reference strain WHO-SRS) to spinosad in feeding bioassays, which was considered to reflect the natural variation of spinosad toxicity in field populations [5]. Additional studies with insecticide-resistant laboratory strains showed that there was no cross-resistance to the major insecticide classes in the housefly [5].

Spinosad resistance in the laboratory housefly strain *rspin* [6] selected for high spinosad resistance, is inherited as a recessive trait linked to autosome 1. The resistance in the *rspin* strain does apparently not involve P450-mediated metabolism as resistance level is unchanged upon pretreatment with the xenobiotic metabolism inhibitor piperonyl butoxide (PBO) [6]. Likewise, spinosad resistance in a Chinese housefly strain was autosomal, although incompletely dominant, controlled by multiple genes and not influenced by PBO [7].

Spinosad resistance in the multi-resistant Danish field population (791a) has been shown to be associated with P450 activity in a study implementing PBO based bioassays as well as gene expression studies [8]. In the spinosad-selected strain 791spin strain, originating from 791a, the male determining factor are located on autosome III [9]. The housefly *CYP4G2* is also located on chromosome III [10]. Insect CYP4G is remarkable by having orthologs distributed across Insecta [11]. The CYP4G enzymes function as oxidative decarbonylases, catalysing the terminal step in insect hydrocarbon production synthesizing alkanes making up a large part of the cuticular hydrocarbons [12].

If spinosad influence expression of xenobiotic genes including P450 genes, we hypothesize that variation of this response is a selectable trait in adaptation to an environment influenced by spinosad, thus having a resistance potential. Additionally, the coordination of P450 gene expression in response to spinosad can elucidate important new insights into the general response to a xenobiotic compound. This paper thus investigates the constitutive and spinosad-induced expression levels of nine resistance associated housefly P450 genes. CYP6A1 assists in the metabolism of organochlorine- and organophosphate-insecticides [13], [14] and is constitutively over-expressed in an organophosphate-resistant strain [15], [16] as well as in neonicotinoid-resistant strains [17]. *CYP6A36* is constitutively overexpressed in a permethrin-resistant strain, whereas no difference was found for *CYP6A37* [18]. *CYP6D1* is constitutively over-expressed in pyrethroid-resistant houseflies [19], [20] and in neonicotinoid-resistant strains [17]. *CYP6D3* is constitutively over-expressed in pyrethroid-resistant houseflies and previously reported involved in insecticide resistance in the housefly [20]. *CYP6G4* is the potential housefly orthologue of *Drosophila melanogaster CYP6G1*, where over-expression is correlated with DDT and neonicotinoid resistance [21], [22]. The mitochondrial CYP12A1 and CYP12A2 metabolize a variety of insecticides and xenobiotics and are constitutively over-expressed in the diazinon-resistant Rutgers strain [23].

## Methods

### Houseflies

Housefly breeding followed standard laboratory conditions. Egg laying was performed on crumpled filter paper soaked in whole milk. Breeding jars (5 L plastic buckets) containing 4 L of medium were seeded with 200 mg of eggs, corresponding to 2700 eggs. The breeding medium consisted of wheat bran 400 g, lucerne meal 200 g, bakers yeast 10 g, malt extract 15 mL, whole milk 500 mL and water 500 mL. For adult feeding, cube sugar and water were given continuously. Feeding started after emergence with whole-milk powder mixed with icing sugar (1∶1 w/w) [24].

The insecticide-susceptible standard reference strain WHO-SRS was received in 1988 from the Department of Animal Biology, University of Pavia, Italy.

The unselected neonicotinoid resistant laboratory population 766b was collected in 2005 in Denmark. Female and male 766b flies were 140-fold and 130-fold resistant to imidacloprid at LC_50_, respectively [25]. The 766b strain is susceptible to spinosad [8].

The multi-resistant 791a laboratory population was collected in the context of a resistance survey in 1997 in Denmark. The strain has never been selected after collection. The strain was highly resistant to pyrethroid, anti-cholinesterase and showed some resistance to the chitin synthesis disrupting larvicides as well as fipronil [26]–[28]. Female and male 791a flies were 20-fold and 22-fold resistant to imidacloprid at LC_50_, respectively [17]. 791a females were 27-fold spinosad-resistant at LC_50_, whereas 791a male houseflies were susceptible (5-fold resistant) [8].

The spinosad selected 791spin strain was created by selection of the spinosad resistant field strain 791a. The initial selection of 791spin was made by 24 h non-choice feeding sugar impregnated with 71 µg spinosad g^−1^ sugar; males (*N* = 573, 9% survival) and females (*N* = 406, 32% survival). Selection was repeated in generations 2, 5, 7, 10, 13, 18 and 22 after the initial selection, with increasing concentrations of spinosad. 791spin females were 21-fold spinosad-resistant at LC_50_, whereas 791spin male houseflies were 6-fold resistant which is considered to be within the natural variation in spinosad toxicity in susceptible Danish field populations [5]. Female and male 791spin flies were considered susceptible to imidacloprid having 3-fold and 4-fold resistance factors at LC_50_, respectively [8]. The strain is retained by regular annually selections with spinosad-impregnated sugar: male flies 0.4 mg spinosad per g sugar and female flies 3.2 mg spinosad per g sugar, for a maximum 72 hours.

The field population 845b (unselected) was collected August 23. 2011 at a dairy farm located at Nykøbing Mors, Denmark (56°53′51.07″N, 8°48′42.81″E). The flies were collected on private land with consent of the owner. The field collection did not involve endangered or protected species. The strain could be characterized as a good representative for Danish field populations with no or a low level of resistance towards neonicotinoids and pyrethroids [29]. F_1_ and F_2_ flies from the 845b field population were used for experiments testing resistance level as well as the influence of spinosad on expression on P450 genes.

### Treatment of houseflies for gene expression analysis

Five to seven days old, adult male and female flies were subjected to a non-choice feeding test with spinosad (88%, 76.1% spinosyn A and 11.9% spinosyn D, DOW AgroSciences). The insecticide were diluted with analytic-grade acetone and impregnated on. Susceptible male and female flies were given spinosad in dose 0.063 mg per g sugar and male and female flies from the field populations 766b and 845b were given spinosad in dose 0.25 mg and 0.5 mg per g sugar, respectively. Males and females of multi-resistant strain 791a were given respectively 0.5 mg and 2 mg spinosad per g sugar [17].

All flies had access to water, milk and sugar *ad libitum* before trials. Flies, which were alive at day 5 (130–500 flies) were placed in cages with full access to water and were given excess of spinosad-treated granular sugar in a small petri-dish as the only food. The feeding tests were carried out at 25–26°C, 60–65% RH in continuous light. Twenty-four hours upon test start living and fresh looking flies were collected by vacuum suction, immediately sedated by cold and killed at –20°C. The flies were hereafter kept on –80°C until RNA extraction.

Parallel experimental set-ups were performed with resistant and susceptible flies, with the one exception that the sugar offered were left acetone-coated. These flies served as reference for examinations of constitutive gene expression levels.

### Primer designs for quantitative real-time PCR

Gene specific primer pairs were designed based on sequences obtained from the NCBI GenBank with the exception of *CYP4G2*, which was extracted from Zhu *et al*. [10]. Gao *et al*. [30] mentioned *CYP6G4* (*Musca domestica*) to be an ortholog to *CYP6G1* (*Drosophila melanogaster*). The *CYP6G1* gene was blasted against the GenBank database (blastn, http://blast.ncbi.nlm.nih.gov/) with 65% identity.

*CYP4G2*, F: 5′-cgaggaggatgatgaaataagcaagc-3′, R: 5′-ttggacatggccatcatggcatct-3′; *CYP6A1* (GenBank: M25367), F: 5′-aattttgccaatcgtggtctg-3′, R: 5′-tccaccattaccaagtggcc-3; *CYP6A36* (DQ642009), F: 5′-aaaggcatggccgttgttat-3′, R: 5′-acttgagaagcggcaaaatg-3′; *CYP6A37* (DQ642010), F: 5′-atgcaaatcctcatccccg-3′, R: 5′-ccgtgactttgtcatgggaga-3′; *CYP6D1* (U22366), F: 5′-gcaaatgcactcaggatttcc-3′, R: 5′-tgcccaagagggagatgataa-3′; *CYP6D3* (AF200191), F: 5′-tgccccataagggaggct-3′, R: 5′-agaccattgactggtactaaaaccg-3′; *CYP6G4* (FJ911556), F: 5′-gctgcaaagcaaattggg-3′, R: 5′-actacgcaccacattcag-3′; *CYP12A1* (U86618), F: 5′-atccgttgaccttgggaaatg-3′, R: 5′-tcatcctgcagcaaacctgtt-3′; *CYP12A2* (U94698), F: 5′-cctatttgagggcctgcat-3′, R: 5′-tgggaacacgatagccact-3′; *GAPDH* (DQ386609) F: 5′-ccggtatctccctcaacg-3′, R: 5′-tgacacggttggagtaaccga-3′.

The primer pairs used were designed not to span introns since the present study used gDNA for external standards in real-time PCR runs. To avoid non-specific amplification all RNA samples were routinely treated with DNase before use. Upon optimization forward and reverse primers were used in optimal concentration 150 nM. Amplicon sequence specificity was verified dissociation curves giving rise to single peaks at the specific melting temperature of the products. A full summary on primer design, optimization and validation is given by Markussen and Kristensen [17].

### RNA and DNA extraction

Total RNA from whole bodies of houseflies was extracted using the RNeasy Maxi kit (Qiagen, Ballerup, Denmark). Pools of flies (*approx*. 1.2 g equivalent to 60 flies) were thoroughly grinded with liquid nitrogen, a mortar and pestle and homogenized with buffer added β-mercaptoethanol supplied by the RNeasy kit according to the manufacturer’s protocol. Isolated RNA was DNase-treated and concentrated using the RNeasy MinElute kit (Qiagen). Gel electrophoresis and spectrophotometry (Nanodrop; NanoDrop Technologies, Wilmington, USA) was performed to assess the integrity and the concentration of each RNA sample, which was dissolved in RNase-free water and stored at −20°C until use.

Extraction of gDNA used for external standards was performed according to the manufacturer’s protocol for the DNeasy kit (Qiagen). Genomic DNA was stored as stocks of 125 ng µL^−1^ at −20°C corresponding to ∼120,000 copies of a single-copy gene. The mass of the haploid housefly genome (the C-value; http://www.genomesize.com) is ∼1.04 pg therefore 1 ng of gDNA from *M. domestica* contains *approx.* 962 copies of a single-copy gene. A fresh 10-fold serial dilution at five quantities ranging from 125 ng (∼120000 gene copies) to 0.0125 ng (∼12 gene copies) was prepared for each real-time PCR run.

### RT reaction and real-time PCR

First-strand cDNA was synthesized from RNA using the High Capacity Reverse Transcription kit (ABI). In a single RT reaction, 1 µg tRNA was mixed with 10 µL RT buffer, 10 µL random primers [10X], 4 µL dNTPs [25X], 5 µL Multiscribe Reverse Transcriptase [5 U µL^−1^] and RNase free water (Qiagen) to a final volume of 100 µL. The reaction was incubated at 25°C for 10 min for primer-RNA binding, followed by reverse transcription at 37°C for 120 min and 85°C for 5 min.

PCR of 20 µL reactions were performed using 20 ng of the cDNA samples, SYBR Green PCR master mix (ABI) and 150 nM of primers specific for the *CYP4G2*, *CYP6A1*, *CYP6A36*, *CYP6A37*, *CYP6D1*, *CYP6D3*, *CYP6G4*, *CYP12A1*, *CYP12A2* and *GAPDH* genes. All samples and the external standards were run in four technical replicates. The strain variance is accounted for by randomization of the flies selected for RNA purification as well as the number of flies used; approx. 60 per sample in addition to 2–4 biological replicas depending on the availability of houseflies. Absence of gDNA was confirmed through PCR runs of no-RT controls in replicates of two.

The PCR runs were performed on ABI PRISM 7500 HT Sequence Detection Systems with Sequence Detection system software version 1.4 (ABI) initiated by a 2 min activation step at 50°C followed by a polymerase activation step for 10 min at 95°C. Amplification was obtained by 40 cycles of 15 s at 95°C with a 1 min anneal and extending step at 60°C. A final dissociation stage at 95°C for 15 sec, 60°C for 15 sec and 95°C for 15 sec was added to generate a melting curve for verification of amplification product specificity. The PCR data in Table 1 and 2 are presented as the mean copy number per 20 ng of RNA ± standard deviation of 2–4 biological replicates. Statistical analysis was undertaken using pairwise Wilcoxon Ranking test, where a P-value less than 0.05 was considered to be statistically significant [31].

**Table 1:** Constitutive and spinosad induced P450 gene expression of a spinosad-susceptible housefly strain (WHO-SRS) and spinosad -resistant housefly strain (791spin) measured by quantitative real-time PCR.

Strain	Gene	Male				Female				Constitutive	Spinosad
		Constitutive	Spinosad	Fold- change	P-value^a^	Constitutive	Spinosad	Fold- change	P-value^a^	P-value^b^	P-value^b^
**WHO-SRS**	*CYP4G2*	947±517	1,810±611		0.1014	2,540±1,858	16,680±508	6.6	0.0050	0.1797	0.0034
	*CYP6A1*	8.9±4.3	25.9±3.1	2.9	0.0006	1.9±1.2	3.2±0.3	1.7	0.0411	0.0012	0.0012
	*CYP6A36*	6.2±1.1	7.2±1.8		0.3829	3.9±1.8	8.4±5.0		0.0593	0.0734	0.8665
	*CYP6A37*	118±44.9	310±22.9	2.6	0.0034	53.3±5.2	141±18.1	2.6	0.0007	0.0034	0.0007
	*CYP6D1*	1,090±331	1,730±1007		0.9999	284±58.3	1,320±174	4.7	0.0003	0.0012	0.9999
	*CYP6D3*	69.4±11.0	206±45.9	3.0	0.0003	13.5±4.7	57.9±19.7	4.3	0.0006	0.0006	0.0003
	*CYP6G4*	154±35.5	394±128	2.6	0.0012	50.8±22.9	348±133	6.9	0.0043	0.0022	0.4318
	*CYP12A1*	21.6±10.2	12.7±9.8		0.1807	7.0±0.8	13.2±1.8	1.9	0.0003	0.0003	0.9452
	*CYP12A2*	36.4±7.5	41.5±5.5		0.2786	10.9±3.6	22.5±5.0	2.1	0.0006	0.0002	0.0002
	*GAPDH*	4,860±680	8,570±1508	1.8	0.0006	4,550±319	8,700±676	1.9	0.0003	0.5358	0.3829
**791spin**	*CYP4G2*	2,100±497	1,010±318	0.5	0.0021	11,790±7106	7,800±2400		0.6943	0.0014	0.0003
	*CYP6A1*	24.3±8.8	42.0±14.4	1.7	0.0201	6.2±4.0	6.8±3.6		0.5358	0.0008	0.0002
	*CYP6A36*	2.4±0.7	3.8±1.1		0.3717	0.8±0.5	3.2±0.7	4.0	0.0148	0.0003	0.8745
	*CYP6A37*	327±71.7	288±19.7	0.9	0.0019	166±77.1	128±14.1		0.9999	0.0007	0.0003
	*CYP6D1*	797±144	599±274		0.2359	662±569	472±57		0.7104	0.6806	0.9999
	*CYP6D3*	405±49.8	467±83.5		0.2198	51.5±28.1	428±191	8.3	0.0012	0.0001	0.9372
	*CYP6G4*	363±95.5	513±223		0.3148	756±413	485±36.1		0.7012	0.2222	0.7104
	*CYP12A1*	200±48.2	305±21.1	1.5	0.0003	66.4±30.6	224±53.9	3.4	0.0001	0.0006	0.0022
	*CYP12A2*	26.3±6.0	33.2±3.0	1.3	0.0091	18.0±12.0	16.7±4.2		0.9452	0.2561	0.0003
	*GAPDH*	6,610±162	5,810±1238		0.1871	6,750±1089	5,670±721		0.1812	0.3984	0.7206

Mean mRNA transcript copy number×1000 is per 20 ng of total RNA. P-values between comparisons of male and female flies and treatment were obtained by a F-test followed by Student’s Significant difference t-Test. Significant P-values (<0.05) are underlined.

a P-values obtained by comparisons of constitutive and spinosad induced gene expression within strain.

b P-values obtained by comparisons of gene expression between sexes.

**Table 2:** P450 gene expression of insecticide-susceptible and -resistant housefly strains upon sugar (constitutive) and spinosad exposure measured by quantitative real-time PCR.

Gene	Strain	Male				Female			
		Constitutive	Spinosad	Fold- change	P-value^a^	Constitutive	Spinosad	Fold- change	P-value^a^
*CYP6A1*	WHO-SRS	8.9±4.3	25.9±3.1	2.9	0.0006	1.9±1.2	3.2±0.3	1.7	0.0411
	766b	15.6±8.9	33.5±11.5	2.1	0.0148	8.7±0.6	8.7±1.1		0.8357
	791a	0.8±0.4	0.6±0.3		0.3939	0.6±0.3	0.7±0.2		0.5622
	845b	55.4±23.6	49.6±19.9		0.8075	25.9±10.4	10.9±5.0	0.4	0.0021
*CYP6A36*	WHO-SRS	6.2±1.1	7.2±1.8		0.3829	3.9±1.8	8.4±5.0		0.0593
	766b	15.1±5.8	21.2±5.3		0.0721	11.5±5.3	11.4±3.0		0.6126
	791a	148±92.2	188±38.3		0.4452	101±58.2	48.9±8.9		0.2949
	845b	34.4±22.3	48.7±15.8		0.5362	46.3±22.9	53.4±16.2		0.4385
*CYP6A37*	WHO-SRS	118±44.9	310±22.9	2.6	0.0034	53.3±5.2	141±18.1	2.6	0.0007
	766b	199±110	283±70.8		0.3374	95.1±27.1	114±46.4		0.9015
	791a	101±72.7	130±18.5		0.9452	45.8±29.2	35.6±14.9		0.5287
	845b	202±48.5	254±68.9		0.1042	156±36.5	218±54.3		0.1321
*CYP6D1*	WHO-SRS	1,090±331	1,730±1,010		0.9999	284±58.3	1,320±174	4.7	0.0003
	766b	3,480±660	5,810±1,110	1.7	0.0022	1670±250	1,990±519		0.3439
	791a	4,010±2,652	3,910±570		0.9999	2,040±1,320	1,190±607		0.2380
	845b	2,360±1,029	1,910±289		0.6503	1,030±312	1,480±849		0.5787
*CYP6D3*	WHO-SRS	69.4±11.0	206±45.9	3.0	0.0003	13.5±4.7	57.9±19.7	4.3	0.0006
	766b	1,220±441	2,020±477		0.0649	495±79.8	645±38.2	1.3	0.0043
	791a	214±154	249±61.1		0.9999	122±94.6	30.4±12.8		0.6691
	845b	846±612	448±143		0.6718	426±107	436±65.2		0.7345
*CYP6G4*	WHO-SRS	154±35.5	394±128	2.6	0.0012	50.8±22.9	348±133	6.9	0.0043
	766b	1,060±246	2,220±293	2.1	0.0034	783±120	378±141		0.5887
	791a	157±130	339±161		0.1014	177±160	58.3±41.3		0.5338
	845b	1,070±762	685±416		0.3845	519±323	893±488		0.2561
*CYP12A1*	WHO-SRS	21.6±10.2	12.7±9.8		0.1807	7.0±0.8	13.2±1.8	1.9	0.0003
	766b	63.2±19.5	129.1±33.6	2.0	0.0041	58.9±11.1	59.9±9.8		0.9551
	791a	64.8±7.7	73.0±23.2		0.7789	45.8±15.5	29.6±3.9		0.1003
	845b	69.7±30.9	38.9±16.0		0.1014	34.9±14.3	62.8±24.0	1.8	0.0020
*CYP12A2*	WHO-SRS	36.4±7.5	41.5±5.5		0.2786	10.9±3.6	22.5±5.0	2.1	0.0006
	766b	124±43.9	344±119	2.8	0.0006	65.5±8.3	313±96.2	4.8	0.0003
	791a	83.7±10.8	65.8±7.9	0.8	0.0093	34.3±13.3	29.2±8.9		0.8286
	845b	110±56.7	99.3±20.5		0.9551	50.0±22.3	87.2±44.5		0.1893
*CYP4G2*	WHO-SRS	947±517	1,810±611		0.1014	2,540±1,860	16,680±508	6.6	0.0050
	766b	10,550±1,444	17,190±4,200	1.6	0.0087	15,040±1,830	21,450±2,390	1.4	0.0065
	791a	12,770±671	5,520±725	0.4	0.0021	18,960±9,490	6,440±1,630	0.3	0.0006
	845b	3,660±2,659	4,640±1,500		0.4598	7,900±4,000	9,530±1,910		0.1589
*GAPDH*	WHO-SRS	4,860±680	8,570±1,510	1.8	0.0006	4,550±319	8,700±676	1.9	0.0003
	766b	9,550±662	10,930±3,680		0.9999	7,460±546	7,430±1,060		0.6126
	791a	11,430±4,981	10,820±1,120		0.7012	10,140±4,210	7,060±1,320		0.9999
	845b	3,780±918	4,670±1,220		0.1673	4,560±1,060	5,090±534		0.1501

Mean mRNA transcript copy number×1000 is per 20 ng of total RNA. P-values between comparisons of treatments were obtained by a F-test for determination of variance differences followed by Welch Significant difference Test. Significant P-values (<0.05) are underlined.

a P-values obtained by comparisons of constitutive and spinosad induced gene expression within strain.

## Results and Discussion

The xenobiotic response of P450s is known to play an important role in the development of insecticide resistance. Enhancement of insecticide detoxification has been associated with increased P450 expression of genes leading to resistance [11]. The present experimental setup was selected in order to elucidate which, if any, of the nine P450 genes associated with resistance in the housefly could be responsible or partly responsible for spinosad resistance in the 791spin strain.

The P450s in this study were selected based on the documentation of their involvement in resistance and/or degradation of xenobiotics. Additionally *CYP4G2* were selected because spinosad resistance possibly is linked to chromosome III [9], where *CYP4G2* is located. The house-keeping gene *GAPDH* was added as a control of expression levels.

The four additional housefly strains of this study comes with different histories; an insecticide-susceptible reference strain (WHO-SRS) kept in laboratory breeding for decades, a neonicotinoid-resistant and spinosad-susceptible field population (766b) kept in breeding for 8 years without selection, a multi-resistant (including spinosad) field population (791a) kept in breeding without selection for 15 years and a newly collected spinosad-susceptible field population (845b).

### Gene expression of a house-keeping gene in Danish houseflies

The house-keeping gene *GAPDH* was included in this study to assess the influence of spinosad on general gene expression. For the most part, *GAPDH* gene expression remained constant within resistant laboratory strains (Table 2). The newly-collected 845b strain had a lower level of *GAPDH* gene expression than the other four strains. Short-term spinosad exposure did not alter expression significantly in any of the five strains tested here (P>0.15), with the exception of the insecticide-susceptible WHO-SRS strain. Here expression was increased 1.8- and 1.9-fold in males and females, respectively (P<0.0006).

### 791spin P450 gene expression

**Figure 1:**
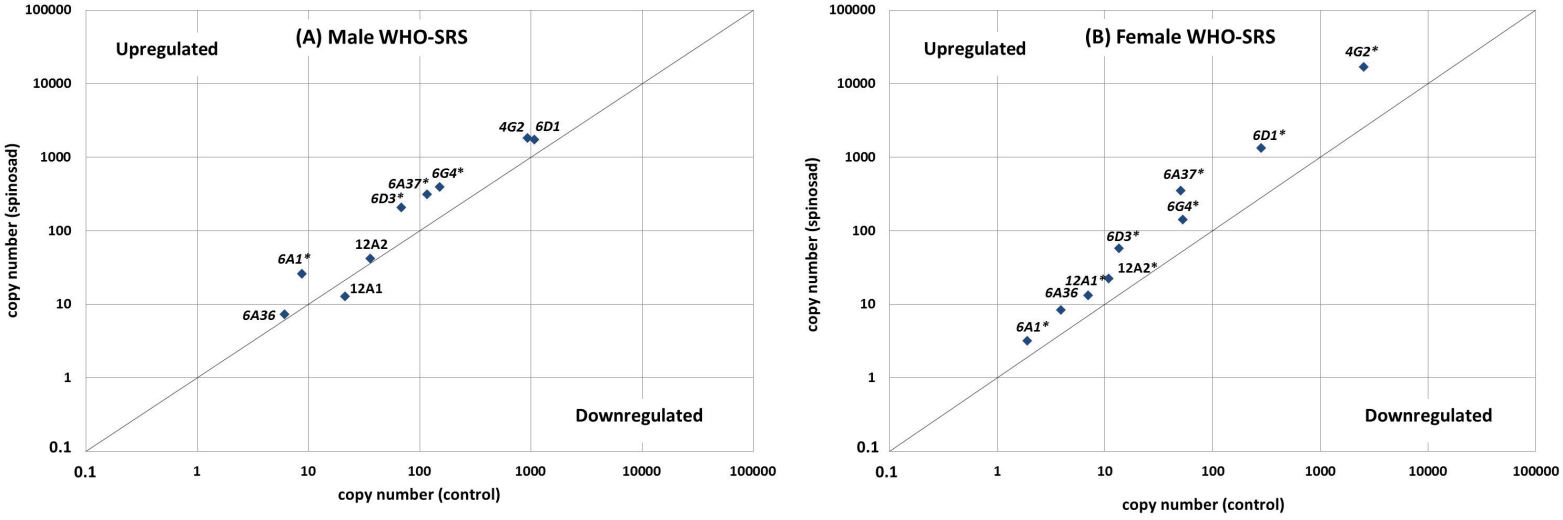
WHO-SRS male (A) and female (B) spinosad-influenced gene expression as a function of the constitutive gene expression. The line represents no effect of spinosad exposure. Genes in right-lower and left-upper corner are down-regulated and up-regulated by spinosad, respectively. Points marked with * are significantly different from the separation line by α = 0.05.

**Figure 2:**
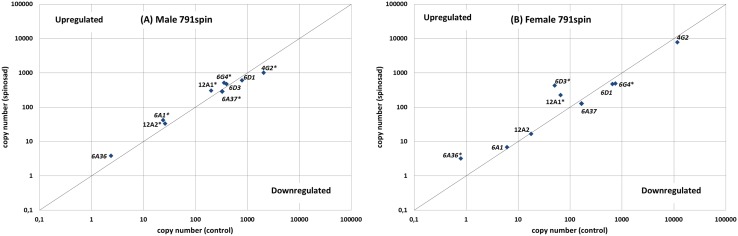
791spin male (A) and female (B) spinosad-influenced gene expression as a function of the constitutive gene expression. The line represents no effect of spinosad exposure. Genes in right-lower and left-upper corner are down-regulated and up-regulated by spinosad, respectively. Points marked with * are significantly different from the separation line by α = 0.05.

A comparison of P450 expression with or without spinosad exposure of 791spin and WHO-SRS showed multiple deviations from neutrality (Figure 1 and 2). Differential expression is mostly up-regulation for both strains, but a few cases of down-regulation were also observed for 791spin (Figure 2). Spinosad exposure in 791spin males caused minor effects (2-fold or less) in both directions. In females, no decreasing effect was observed for spinosad exposure, while the *CYP6A36*, *CYP6G4* and *CYP12A1* were up-regulated 4-, 8.3- and 3.4-fold, respectively (Table 1).

Most of the genes tested here had a higher constitutive expression in 791spin than in the susceptible WHO-SRS strain for both males and females (Table 1). *CYP6A36* had the lowest level of expression of the P450s investigated, while the highest expression was that of *CYP4G2* for both sexes.

The 791spin strain has a sex-dependent resistance profile with regard to spinosad. Females were resistant, while males were considered within the range of susceptibility. This could indicate that the responsible gene(s) would be highly expressed in females compared to males in this strain due to the sex-specific resistance pattern. This was only the case for one single gene, *CYP4G2* (P<0.0014). Sex determination in 791spin is due to a factor on autosome III [9], which led to a hypothesis of the spinosad resistance being due to a factor on the corresponding copy of autosome III. *CYP4G2* is located on autosome III, and is induced by permethrin in the permethrin-resistant ALHF houseflies [18]. Multiple carbon-oxygen bonds, including aldehydes, are present in spinosad [1]. Spinosad or initial degradation products of spinosad could be substrates for the *CYP4G2* enzyme, which is a decarbonylase enzyme catalyzing cleavage of long-chain aldehydes to hydrocarbons with release of CO_2_ [12]. The increased constitutive expression in females compared to males fits with *CYP4G2* as a contributor to spinosad resistance in 791spin. Crossover in male houseflies is rare and it could be suggested that the male factor is connected to a certain low–expressed allele of the *CYP4G2* gene, located on the same chromosome. Then females might have another version of the allele, which is expressed at a higher level. Then females would have two highly-expressed copies of *CYP4G2*, while males have one highly-expressed copy and one low-expressed copy (linked with the M factor). This could explain the sex-specific difference in spinosad resistance.

*CYP6G4* is a possible ortholog of the *CYP6G1* gene in *D. melanogaster* and constitutive overexpression of *CYP6G1* is causing DDT and neonicotinoid resistance in the fruit fly [21], [22]. A similar role for *CYP6G4* in houseflies could be suggested. Recently, *CYP6G4* has shown to be over-expressed in a pyrethroid resistant housefly strain from China, but no causal link was established [30]. In this study, *CYP6G4* expression was higher in the 791spin strain compared to the susceptible strain, 2-fold in males and almost 15-fold in females. However, *CYP6G4* gene expression in 791spin was lower than that of the spinosad susceptible strains. *CYP6G4* gene expression was not significantly different between sexes, regardless of treatment (Table 1). Spinosad treatment caused a gene expression increase in males of the 791spin strain; whereas spinosad treatment decreased *CYP6G4* expression in females, but neither of the effects were significant (P<0.70). This is contradicted by the other spinosad susceptible strains having a higher expression level of *CYP6G4* than 791spin, so a possible role of *CYP6G4* seems to be minor. Further investigations, especially description of the *CYP6G4* alleles, are needed to elucidate the role of *CYP6G4* in this strain as well as its potential role in xenobiotic metabolism and its importance as a housefly insecticide resistance gene.

Constitutive *CYP12A1* expression was 3-fold higher in male houseflies compared to females for both WHO-SRS and 791spin. Furthermore, 791spin flies had an expression level of approximately 10-fold that of WHO-SRS. The sex-dependent difference in *CYP12A1* gene expression remained after spinosad exposure (P<0.0022) in 791spin, but was eliminated in WHO-SRS (Table 1). Spinosad increased *CYP12A1* expression in 791spin males (P<0.0003) and females (P<0.0001) 1.5- and 3.4-fold, respectively (Table 1). The high constitutive expression of *CYP12A1* in 791spin flies could indicate importance in spinosad resistance, but the fact that male expression is 3-fold higher than female constitutive expression indicates the opposite.

In 791spin males, the constitutive *CYP12A2* expression was increased 1.3-fold by spinosad exposure (P<0.0091), while in the resistant females, no effect of short-term spinosad exposure was observed (P>0.95). Expression in 791spin is similar to WHO-SRS expression, and lower than the other strains (Table 1 and 2), indicating no specific role for *CYP12A2* in spinosad resistance in 791spin.

Increased expression of *CYP6A36* has like *CYP6D1* been associated with pyrethroid resistance in the USA, whereas *CYP6A37* did not differ between ALHF and a susceptible strain [10], [18]. No significant effect of spinosad treatment on *CYP6A36* gene expression was observed for any of the five strains (P>0.072), with the exception of 791spin females. Here spinosad caused a 4-fold increase in *CYP6A36* gene expression. Furthermore, 791spin had a low *CYP6A36* expression compared to the other strains of this study irrespective of sex (Table 2). The very low expression of CYP6A36 in 791spin disqualifies *CYP6A36* as responsible for spinosad resistance, while the specific female induction points in the other direction. Spinosad exposure caused a significant decrease (P<0.0019) in *CYP6A37* gene expression for male 791spin flies (Figure 2). Independent of treatment, *CYP6A37* expression was significantly lower in both WHO-SRS (P<0.0034) and 791spin (P<0.0007) females than in males (Table 1). This suggests no role for *CYP6A37* in sex-specific spinosad resistance in 791spin.

The epoxidation activity of the cytochrome P450 CYP6A1 enzyme is possibly linked to organochlorine and organophosphate resistance, and also has juvenile hormone I and III as substrate [32]. The data for *CYP6A*1 expression show a higher expression level in 791spin flies compared to WHO-SRS flies. Spinosad exposure increases expression levels in WHO-SRS 2.9- and 1.7.fold in males and females, respectively (P<0.041). A similar effect is observed for 791spin males, where expression was induced 1.7-fold (P<0.020). However, female expression remained at a similar level (P>0.54). The induction of *CYP6A1* gene expression by spinosad in susceptible WHO-SRS flies, combined with the lack of effect in 791spin females, indicate a possible role for *CYP6A1*. However, in 791spin male constitutive expression is higher than female expression, which indicates no role for *CYP6A1* in 791spin.

Prior results of feeding bioassays and gene expression studies in 791spin and its parental 791a strain suggest an involvement of cytochrome P450 enzymes in housefly spinosad resistance, which seems to be specifically female-linked [8], [17]. These synergist and gene expression studies indicated a minor role of cytochrome P450 genes *CYP6D1* and *CYP6D3* in spinosad resistance in male and female houseflies, respectively. *CYP6D1* is over-expressed in flies resistant to pyrethroids and neonicotinoids [17], [19], [20]. A wide range of substrates is known for the CYP6D1 enzyme including the organophosphate chlorpyrifos and the synthetic pyrethroids deltamethrin and cypermethrin [33]–[35]. *CYP6D3* has been used as a reference to *CYP6D1* and is also located on chromosome I [36]. In this study, male constitutive expression of *CYP6A1* and *CYP6D3* was higher in 791spin than in WHO-SRS flies. Likewise, female constitutive expression of *CYP6A1* was also higher in resistant flies compared to susceptible. This correlates with the pervious study of *CYP6A1*, *CYP6D1* and *CYP6D3* [8]. However, here male *CYP6D1* and female *CYP6D3* expression in 791spin did not differ from WHO-SRS. Markussen et al. (2012) suggested a role for *CYP6D1* and *CYP6D3* in male and female resistance, respectively. However, here the opposite is indicated by a high *CYP6D1* and *CYP6D3* expression in 791spin females and males, respectively. This further complicates the determination of the importance of these two genes in spinosad resistance.

Increased expression has been linked to elevated resistance by increased enzymatic degradation of insecticide e.g. degradation of deltamethrin by CYP6D1 [19], [20]. But, what does down-regulation signify? It could be hypothesized that down-regulation is due to reduced energy costs. Perhaps down-regulation is a part of a coupled system, so that groups of P450 genes are up-regulated and others are down-regulated. An example of this is a transcription factor pathway in Drosophila where 20% of differential expressed genes (including multiple resistance associated P450s) are genes targeted by the transcription factor CncC [37].

Alternatively the action is more direct and a given P450 enzyme activates the insecticide, making it more toxic. This is e.g. shown by bioassay experiments with neonicotinoids. The addition of the synergist PBO to feeding test with imidacloprid increased toxicity 9-fold, whereas toxicity of thiamethoxam decreased in male houseflies [17]. Thiamethoxam has also been shown as a pro-insecticide in plants [38]. Down-regulation will thus have a direct survival effect.

### Effect of spinosad in a new field strain

The field population 845b was included in this study to examine a neonicotinoid- and spinosad-susceptible field population. In general, high levels of P450 gene expression was found in this newly acquired field population for eight out of nine P450 genes in both males and females. Female expression of *CYP6D3* was more than 30-fold higher in 845b than in WHO-SRS (Table 2). This was despite 845b proving susceptible to pyrethrin synergized by PBO, imidacloprid and spinosad by test with discriminating doses [29]. Short-term spinosad exposure had no effect on gene expression of P450 genes with a few exceptions. In females, *CYP6A1* expression was decreased 2.5-fold (P<0.0021) and *CYP12A1* expression was increased 1.8-fold (P<0.0020). None of the other seven P450 genes were affected by spinosad in females (P>0.13). Furthermore, spinosad caused no effect in 845b males for any of the nine P450 genes tested here (P>0.10).

The high gene expression levels observed in this newly-collected strain could be due to the energy requirements involved in maintaining an alert detoxification system. The 845b strain still represent life in the field, while the other strains used for this study have adapted to the laboratory, properly losing some of the defense mechanisms involved in being a wild fly. The pattern of decreased expression in strains adapted to the laboratory was observed for the 845b strain in a two-year study of gene expression [39].

### Effect of spinosad in an insecticide-susceptible reference strain

The highest constitutive P450 expression in the susceptible reference strain WHO-SRS was observed for *CYP4G2* in both sexes, whereas *CYP6A36* and *CYP6A1* were the lowest expressed genes (Table 1).

In general, spinosad exposure caused an increase in gene expression of the nine P450 genes described here for both males and females (Figure 1). *GAPDH* was doubled by short-term spinosad exposure (Table 1). When taking that into consideration and normalizing to *GAPDH* levels, only a few P450 genes were affected by spinosad. In males, the induction of gene expression was no higher than 3-fold with the non-normalized data, while female induction ranged from 1.7-fold to almost 7-fold. In no case did spinosad exposure decrease the expression level significantly.

*CYP4G2* expression was increased 6.6-fold by spinosad in females (P<0.0050). A similar increase (P<0.0043) was observed in expression of *CYP6G4*, while *CYP12A1* and *CYP12A2* were increased approximately 2-fold in females (P<0.0006).

Furthermore, spinosad caused an increase in expression of *CYP6A1* (P<0.041), *CYP6A37* (P<0.0034) and *CYP6D3* (P<0.0006) for both males and females. However, no effect of spinosad was observed for male *CYP6D1*, *CYP12A1* and *CYP12A2* expression as well as for *CYP6A36* expression.

The most remarkable effect by spinosad was observed for *CYP6G4*, where WHO-SRS female expression was increased almost 7-fold (P<0.0043) and male expression 2.6-fold (P<0.0012) as a result of short-term spinosad exposure. This could indicate a role for *CYP6G4* in spinosad resistance or at least a role in xenobiotic response. In most cases, gene expression of P450 genes was induced in the susceptible strain, suggesting spinosad affecting several genes, not all responsible for the resistance mechanism.

### Effect of spinosad in a neonicotinoid-resistant laboratory strain

In the neonicotinoid-resistant laboratory population 766b the highest constitutive gene expression was observed for *CYP4G2* and *CYP6D1*, for both males and females (Table 2). Noteworthy is the 7-fold and 15-fold higher constitutive expression of *CYP6G4* in males and females, respectively, compared to WHO-SRS. The 766b female *CYP4G2, CYP6D3* and *CYP12A2* expression increased (1.4- to 4.8-fold), upon spinosad treatment (P<0.0043). Male *CYP6D3* gene expression was not affected by spinosad (P>0.065), while *CYP4G2* and *CYP12A2* expression increased in females 1.6-fold (P<0.0087) and 2.8-fold (P<0.0006), respectively. Furthermore, *CYP6A1*, *CYP6D1* and *CYP6G4* were induced in males (P<0.015).

Furthermore, spinosad treatment caused gene expression in males to increase 2-fold for *CYP12A1* (P<0.0041), whereas females didn’t change *CYP12A1* expression (P>0.96).

*CYP6D1* appears to be involved in male neonicotinoid resistance while female resistance seems to be linked to over-expression of the *CYP6D3* gene [17]. Both *CYP6D1* and *CYP6D3* expression proved higher in 766b than in WHO-SRS. *CYP6D1* in males was induced by spinosad, while *CYP6D3* was induced in females, indicating possible roles for these genes in resistance.

The high *CYP6G4* gene expression observed in 766b in comparison with WHO-SRS could indicate a role of *CYP6G4* in 766b neonicotinoid resistance, but not in spinosad resistance, since 766b is susceptible to spinosad. Additionally, previous studies has shown that neonicotinoid resistance in strain 766b responded to PBO, indicating metabolism associated resistance as well as reduced expression of the nicotinic acetylcholine-receptor subunit Mdα2 [17], [40].

### Effect of spinosad in a multi-resistant laboratory strain

In the multi-resistant laboratory strain 791a the *CYP6A1* gene was expressed at the lowest level of the genes described in this strain, whereas *CYP4G2* expression was expressed at the highest level, regardless of treatment (Table 2). Spinosad decreased *CYP4G2* gene expression (P<0.0021). Likewise, the *CYP12A2* gene was decreased by spinosad treatment in males, while the other seven genes were unaffected by spinosad (P>0.10).

## Conclusion

It can be concluded that there is very clear difference between the expression of P450 genes in male compared to female houseflies. Most of the genes tested where spinosad caused an effect were up-regulated by spinosad exposure. In the present investigation a marked difference between a true field strain (F_1_) and the laboratory reared strains was observed.

Four requirements were purposed to help identify possible resistance genes in this study; 1) higher constitutive expression in 791spin compared to WHO-SRS, 2) spinosad induction of P450 expression in WHO-SRS or other spinosad-susceptible strains, while 3) no significant effect was observed in 791spin, 4) female constitutive expression in 791spin is higher than male expression.

*CYP4G2*, *CYP6A1*, *CYP6D3* and *CYP6G*4 have an expression pattern somewhat fitting the requirements and therefore the resistance profile of 791spin, but only *CYP4G2* fit all four requirements significantly, making it the most likely candidate. The overall high expression level of *CYP4G2* throughout the strains also indicates importance of this gene. However, the data on 791spin are not conclusive concerning spinosad resistance and e.g. pointing to a single spinosad resistance gene. Small contributions from multiple P450s with different enzymatic capabilities could be speculated to do the job in 791spin.

Analyzing the expression of metabolic detoxification genes rarely gives a clear and unambiguous answer to which enzymes are involved in resistance [41]. Furthermore, the P450s ability to attack the highly complicated spinosad molecule is still unresolved. There is an indication of *CYP6G4* as an insecticide-resistance gene, where involvement in spinosad resistance cannot be rejected. The high expression levels of P450 genes in flies from a field population compared to established laboratory strains presented in this study questions the use of laboratory strains beyond resistance gene identification and warrants studies of P450 expression in field populations, since only the heterogeneous nature of field populations would allow for the selection of the rare variants corresponding to resistance alleles likely to trigger control failure [42]. Laboratory strains can only be used for assessing field control issues if the starting genetic variation is present in the field strain adapted to the laboratory. Differential expression is both up- and down-regulated. Increased expression has been functionally linked to elevated resistance, but it is still puzzling what down-regulation signifies. Is it a part of an interconnected regulatory network? Is it linked to the overall energy budget? Or is it linked to toxicology dynamics of the P450 system?

The differences in expression of minor and major insecticide resistance genes are some of the important tools in pesticide resistance management aiming to limit or prevent development of resistance by controlling factors, which may lead to resistance. This study serves as a stepping stone for the dissection of P450 expression in houseflies in relation to xenobiotics.
